# Chinese Propolis Prevents Obesity and Metabolism Syndromes Induced by a High Fat Diet and Accompanied by an Altered Gut Microbiota Structure in Mice

**DOI:** 10.3390/nu12040959

**Published:** 2020-03-30

**Authors:** Yufei Zheng, Yuqi Wu, Lingchen Tao, Xi Chen, Trevor Joseph Jones, Kai Wang, Fuliang Hu

**Affiliations:** 1 College of Animal Sciences, Zhejiang University, Hangzhou 310058, China; iriszheng92@gmail.com (Y.Z.); hzhzwuyuqi@163.com (Y.W.); 11817025@zju.edu.cn (L.T.); 21717031@zju.edu.cn (X.C.); 2 Department of Radiation Oncology, Helen Diller Family Comprehensive Cancer Center, University of California, San Francisco, San Francisco, CA 94115, USA; Trevor.Jones@ucsf.edu; 3 Institute of Apicultural Research, Chinese Academy of Agricultural Sciences, Beijing 100093, China; kaiwang628@gmail.com

**Keywords:** Chinese propolis (CP), obesity, high-fat diet, metabolic syndromes, microbiome

## Abstract

The increasing incidence of obesity poses a great threat to public health worldwide. Recent reports also indicate the relevance of obesity in metabolic diseases. Chinese propolis (CP), as a well-studied natural nutraceutical, has shown a beneficial effect on alleviating diabetes mellitus. However, few studies have investigated the effect of CP on weight management and energy balance. We examined the beneficial effects of dietary CP on weight in high-fat diet-fed female and male mice and determined whether CP alters gut microbiota. In this study, dietary CP supplementation reduces body weight and improves insulin resistance in high-fat diet (HFD)-fed mice in a dose-dependent manner. CP treatment also reverses liver weight loss and triglyceride accumulation in association with hepatic steatosis. The 16S rRNA analysis of gut microbiota demonstrated that CP treatment modulates the composition in HFD-fed mice. Our study also suggests that male mice were more sensitive to CP treatment than female mice. Taken together, CP supplementation reduces weight gain and reverses gut microbiome dysbiosis induced by HFD. Further, the effects of CP treatment on metabolic biomarkers and microbiome structure differ by gender.

## 1. Introduction

Obesity is a worldwide public health threat that is increasing among adults and youth [1]. A long-term high-fat or unbalanced diet contributes to obesity [2,3], which is reflected by the increasing global overweight ratio [4]. The incidence of obesity has reached 108 million children and 604 million adults in 2015, and the prevalence has doubled since 1980 [1]. According to a recent epidemiological survey, 37% of men and 38% of women in USA are estimated to have a body mass index (BMI) greater than 25 kg/m^2^, which is considered as obesity [5]. High BMI contributed to 3.5 million deaths in 2010 and is the fifth leading cause of death in the world. It is also associated with the increased incidence of cancer, metabolic syndromes, and cardiovascular diseases [6]. Reports have pointed out that the significant increase in the prevalence of obesity leads to a rapid increase in type 2 diabetes, which poses a great threat to public health [7]. Therefore, it is urgent to find methods to help manage the weight gain and complications induced by obesity.

Some scientists believe natural products offer an alternative method to exercise training that can help control the fat accumulation induced by an unhealthy diet. In a recent study, natural products, especially those rich in polyphenols, have shown great therapeutic value for the prevention and treatment of obesity via inhibiting adipocyte lipid accumulation [8]. Anhe et al. revealed that a polyphenol-rich cranberry extract could prevent diet-induced obesity and insulin resistance as well as alleviate intestinal inflammation [9]. Lama reported polyphenol-rich virgin olive oil could improve mitochondria dysfunction and non-alcoholic fatty liver disease (NAFLD) in rats fed a high-fat diet [10]. Propolis, high in polyphenols and flavonoids, is a resinous substance collected by bees from plants exudates that is mixed with wax and mandibular gland secretions [11]. Propolis has long been recognized as a natural nutraceutical by exerting great anti-inflammatory, anti-bacteria and anti-oxidant effects [11]. Studies have reported that propolis extract could boost lipid metabolism, alleviate insulin resistance, and delay obesity in high-fat diet-fed mice and rats with type 2 diabetes [12,13]. Additionally, ethanol-extracted propolis can regulate blood glucose, glucose metabolism, and blood lipid concentration in rats with diabetes mellitus [14]. A major component in propolis, caffeic acid phenethyl ester, has an anti-obesity effect on high-fat diet-induced adipogenesis at a very early stage by regulating cyclin D1 [15].

Studies have indicated that intestinal microbiota have various effects on human weight in response to different diets [16,17]. Changes in intestinal bacteria are believed to play a decisive role in regulating energy balance, glucose, lipid metabolism and chronic inflammation related to obesity [18,19]. These bacteria participate in digestive processes, energy regulation, vitamin synthesis, protection against pathogenic microorganisms, and modulation of the immunologic system [20]. Meanwhile, growing evidence has associated microbiota modulation with obesity [21]. It was suggested that certain phyla, classes, or species of bacteria could be beneficial or detrimental to obese patients. Thus, it would be promising to develop strategies for changing gut microbiota to manage obesity and obesity-related disorders [22]. Wang et al. has discovered that Chinese propolis (CP) could alleviate dextran sulfate sodium-induced colitis in rats fed a Western diet by reshaping microbiota composition [23]. Roquetto et al. tested green propolis on high-fat diet-fed mice, finding that propolis reversed the elevation of *Firmicutes* and inflammatory biomarkers expression induced by diet in the obese mice [24]. Here, we tested whether CP has positive effects on the composition of gut microbiota in high-fat diet-fed mice. To determine whether CP has protective effects on the gut symbiont, we correlated the anti-obesity and beneficial metabolic effect of CP administration on high-fat diet-fed mice with the composition of the intestinal microbiota.

## 2. Materials and Methods

### 2.1. Animals and Experimental Design

Female and male C57BL/6 6–8-week-old mice were obtained from Zhejiang Chinese Medicine University and maintained in a special pathogen-free condition. Mice were randomized by gender (20 ± 0.6 g for female, 27 g ± 0.5 g for male) to be fed a normal diet (ND), high-fat diet (HFD), high-fat diet with low-dose propolis (LP), high-fat diet with high-dose propolis (HP) for 9 weeks. LP and HP mice were treated with 150 and 300 mg/kg propolis, respectively, by gavage daily for 9 weeks. We use these doses according to the preliminary experiment. Body weight was measured every week.

The CP used was well characterized in our prior study (Table S1) [25]. The HFD (60% kcal fat, D12492)) and ND (10% kcal fat, D12450) mouse chows were purchased from Research Diet Inc. (New Brunswick, NJ, 08901, USA) and stored at −20 °C.

This study was conducted in accordance with the Declaration of Helsinki, and the protocol was approved by the Ethics Committee of Zhejiang Chinese Medical University, No.SYXK, Zhejiang, 2013–0164, China.

### 2.2. Histological Analysis

Epididymal white adipose tissue (Epi-WAT), parametrial white adipose tissue (Par-WAT), perirenal white adipose tissue (Per-WAT), mesenteric white adipose tissue (Mes-WAT), inguinal subcutaneous adipose tissue (Ing-SAT), and brown adipose tissue (BAT) were collected at autopsy upon experiment termination at 9 weeks. In addition, liver, small intestine and blood were collected.

Frozen liver sections were stained with Oil Red O. Epi-WAT, BAT, and small intestine were fixed in 4% paraformaldehyde, paraffin-embedded and sectioned at 4 μM. Hematoxylin and eosin staining were performed according to the standard protocol for pathological analysis.

### 2.3. Biochemical Parameters

Serum samples were collected from the heart and separated by centrifugation at 1000 g for 10 min at 4 °C. Hitachi high-technologies global 7020 clinical analyzer was used to test serum biochemical parameters. The concentrations of triglycerides (TGs), cholesterol (CHOL), high-density lipoprotein (HDL), low-density lipoprotein (LDL), glucose, alanine aminotransferase (ALT) and aspartate aminotransferase (AST) were measured by DiaSys diagnostic systems (Shanghai, 200120, China). The concentration of plasma lipopolysaccharide (LPS) was measured using a Genscript ToxinSensorTM Chromogenic LAL Endotoxin Assay Kit (Jiangsu, China). The concentration of TGs in liver tissue was measured by using an Adipogenesis Kit (Sigma, 14508, USA, MAK040).

### 2.4. Serum ELISA Analysis

Serum was used to measure the levels of inflammatory cytokines. Interleukin-6 (IL-6) and tumor necrosis factor-α (TNF-α) concentrations were measured by an enzyme-linked immunosorbent assay (ELISA) purchased from Elabscience Biotechnology (Wuhan, 430070, China).

### 2.5. Glucose and Insulin Tolerance Analyses

For the intraperitoneal glucose tolerance test (IGTT), during the 8th week of the experiment, mice were fasted for 12 h before receiving an IP injection of glucose (1.5 g/kg). Blood samples were taken from the tail vein at 15, 30, 60, and 120 min post injection, and blood glucose was measured using Accucheck performa (Roche, 6980, Germany). In the 9th week, we administrated the insulin tolerance test (ITT). Mice were fasted for 6 h before receiving an intraperitoneal injection of insulin (0.75 UI/kg). Blood glucose levels were measured after 15, 30, 60, 120, and 180 min. We calculated the homeostasis model index of insulin resistance based on the serum fasting blood glucose and insulin concentrations (HOMA-IR: insulin × glucose/22.5).

### 2.6. Gene Expression Analysis

The RNA of liver tissue, Epi-WAT, and BAT was extracted by RNAprep Pure tissue kit of Tiangen Biotech Co., Ltd. (Beijing, 100192, China) according to the manufacturer’s protocols. The concentration of RNA in the samples was measured by NanoDrop spectrophotometer (ND-2000, NanoDrop Technologies, USA) and stored at −80 °C. We performed cDNA synthesis and qRT-PCR as described in our previous study [25]. The housekeeping gene *GAPDH* was used to normalize the expression of the other target genes, and the results were expressed as 2^−ΔΔCt^. Primers used in this study are listed in the Supplementary Materials Table S2.

### 2.7. Gut Microbiota Analysis

DNA was extracted from fresh feces (300 mg ± 80 mg) collected during the final 3 days of this study. Bacterial genomic DNA was extracted using the CTAB/SDS method. Polymerase chain reaction (PCR) reactions targeting the V3–V4 regions of the 16S rDNA genes used the specific primer with the barcode (341F, 5’-CCTAYGGGRBGCASCAG-3’, 806R, 5’-GGACTA CNNGGGTATCTAAT-3’). The 16S rRNA gene sequences were deposited in NCBI Sequence Read Archive and the corresponding accession numbers of BioProject are PRJNA562777 for male and PRJNA563139 for female.

Paired-end reads from the original DNA fragments were merged using FLASH. Sequences were analyzed using the Quantitative Insights Into Microbial Ecology (QIIME) software package, and in-house Perl scripts were used to analyze alpha (within samples) and beta (among samples) diversity. We used pick_de_novo_otus.py to pick operational taxonomic units (OTUs) by making an OTU table. Sequences with ≥97% similarity were assigned to the same OTUs. We picked a representative sequence for each OTU and used the RDP classifier to annotate taxonomic information for each representative sequence. In order to compute alpha diversity, we rarified the OTU table and calculated three metrics: Chao1, Observed Species, and the Shannon index. Rarefaction curves were generated based on these three metrics.

### 2.8. SCFA Analysis

Colonic digesta collected from mice at the time of autopsy were weighed and diluted with 700 μL deionized water containing 0.92 μg/μL 2-ethylbutyric and then centrifuged at 10,000 g and 4 °C for 10 min. The supernatant fluid was mixed with 25% metaphosphoric acid solution in a ratio of 4:1 at room temperature for 3 h. The mixture was centrifuged at 10,000 g and 4 °C for 10 min and the supernatant was used for determination of short-chain fatty acids (acetic acid, propionic acid, butyric acid, isobutyric acid and isovaleric acid).

Analysis was conducted using the GC-2010 gas chromatography (Shimadzu Corporation, Kyoto, Japan) with a flame ionization detector (250 °C) and an ATOE-FFAP column (30 m × 250 μm × 0.25 μm). In total, 1 μL of sample was injected and the injector (250 °C) worked in the split mode at a ratio of 10:1. The initial column temperature was 60 °C, rising to 220 °C at 20 °C /min. The carrier gas was N_2_ at a speed of 0.8 mL/min.

### 2.9. Statistical Analysis

Results were expressed as the mean ± SEM or the mean ± SD obtained from at least 8 biological replicates. Comparisons between controls and each treatment were performed using Student’s unpaired t-test and marked with * *P* < 0.05; ** *P* < 0.005; *** *P* < 0.001 (Prism 8.0; GraphPad Software, San Diego, CA, USA).

## 3. Results

### 3.1. Chinese Propolis Reduces Weight Gain in HFD-fed Mice

**Figure 1:**
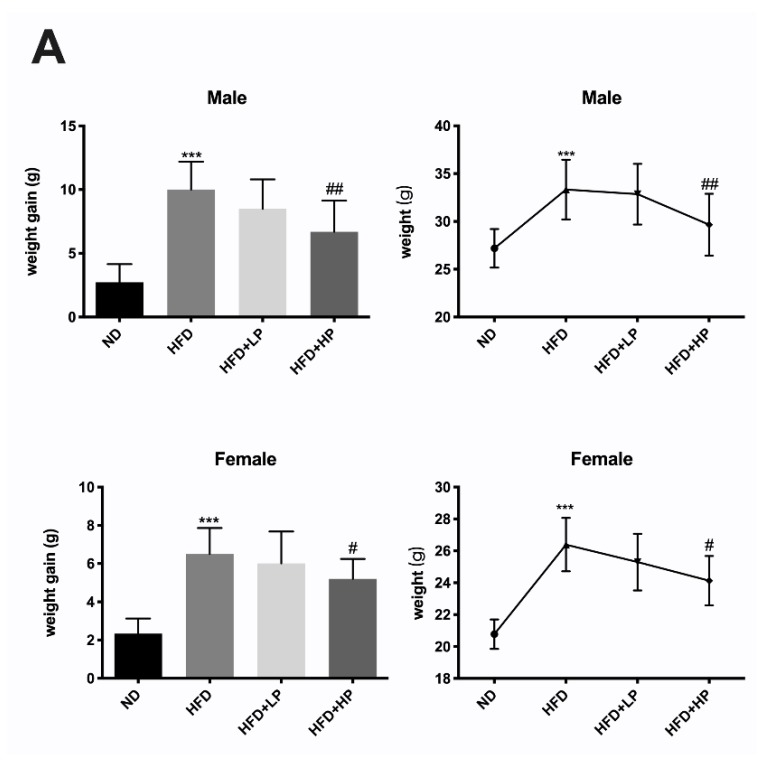

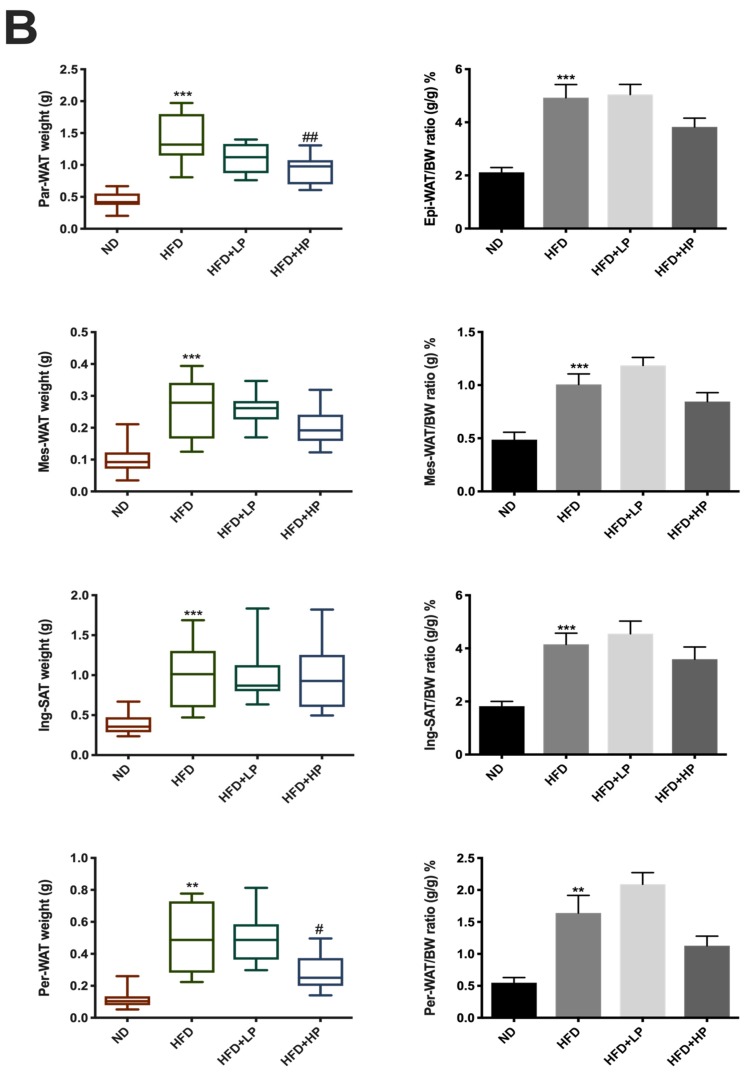

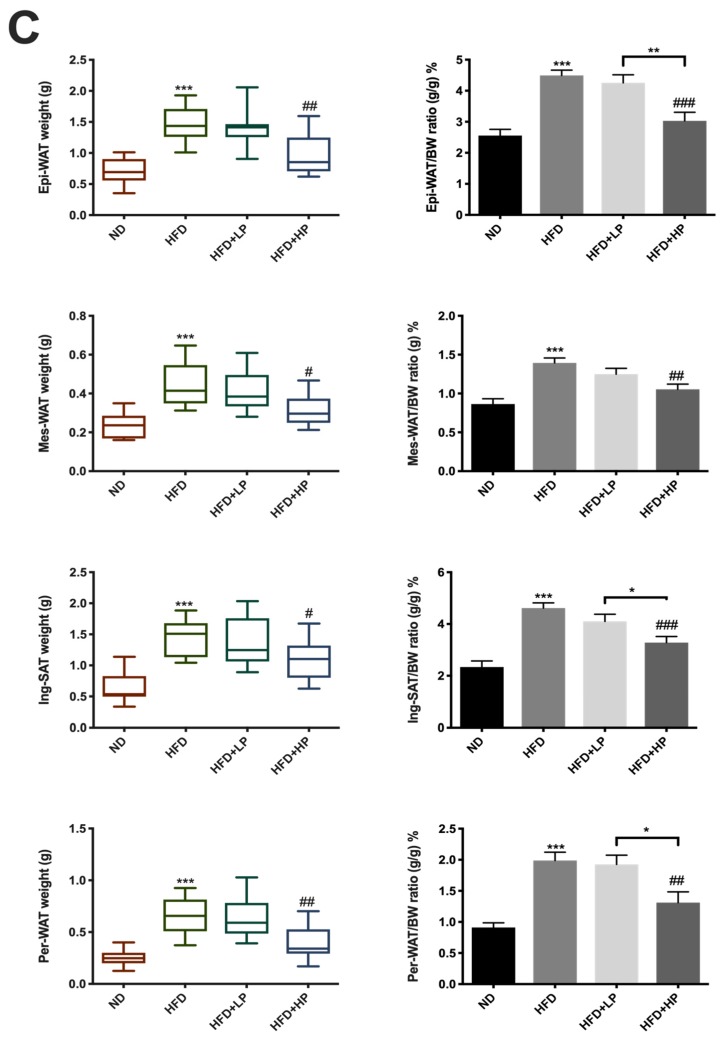
Chinese propolis (CP) prevented body weight gain and fat accumulation in both female and male high-fat diet (HFD)-fed mice. (**A**) The mean weight gain and weight of each group of both genders. (**B**) The weight of parametrial white adipose tissue (Par-WAT), mesenteric white adipose tissue (Mes-WAT), inguinal subcutaneous adipose tissue (Ing-SAT) and perirenal white adipose tissue (Per-WAT) and their ratios to body weight of female mice. (**C**) The weight of Par-WAT, Mes-WAT, Ing-SAT and Per-WAT as well as their ratios to body weight of male mice.

We measured the body weight of female and male mice every week. As expected, the HFD group mice gained much more weight compared to the ND group, regardless of the gender (Figure 1A). The administration of LP failed to prevent HFD-fed mice from becoming overweight, while HP significantly decreased body weight under HFD in both female and male mice (Figure 1A).

Upon termination, the main adipose tissues, including Epi-WAT, Par-WAT, Per-WAT, Mes-WAT, and Ing-SAT were isolated and weighed. As shown in Figure 1B,C, HFD increased the weights of all these types of adipose tissues. The ratio of adipose tissues normalized to body weights of HFD-fed mice was significantly different compared to those on ND (*p* < 0.001). In accordance with the change in body weight, LP did not prevent the accumulation of adipose tissues in both female and male mice. In contrast, HP lowered the adipose tissue weight and the adipose/body weight ratio, especially in male mice (*p* < 0.005). In female mice, there was also a trend toward a decline.

### 3.2. CP Improves Glucose Resistance and Lipid Metabolism in HFD Mice

**Figure 2:**
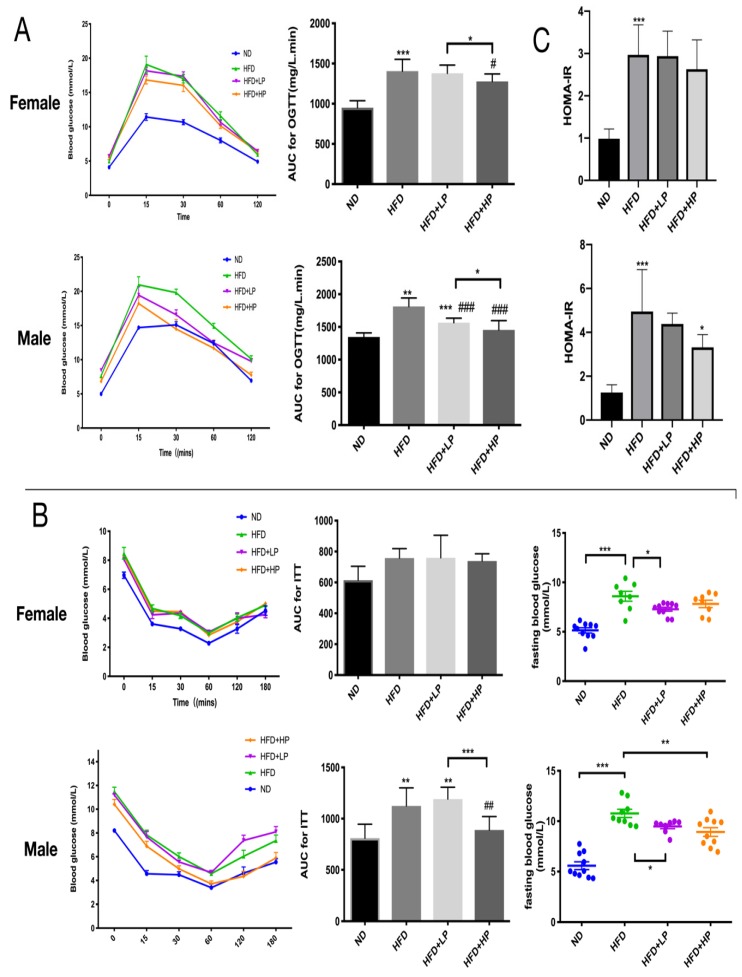

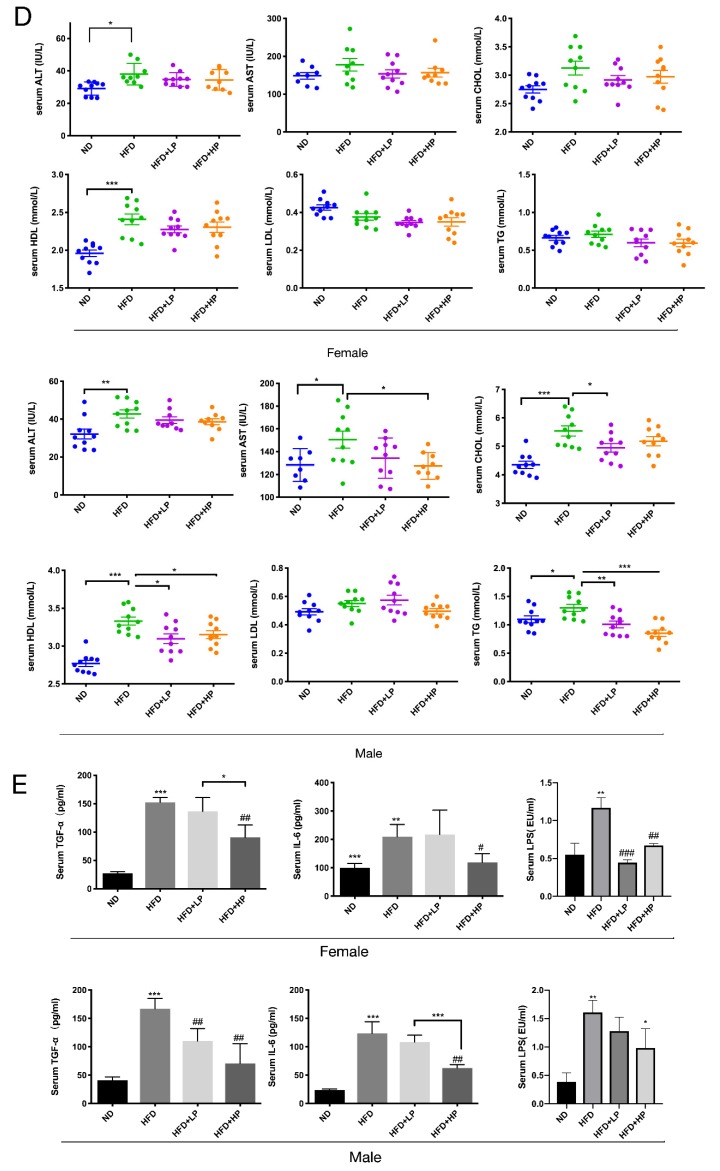
CP administration improved insulin sensitivity and lipid metabolism in HFD-fed mice. Oral glucose tolerance tests (**A**) and insulin tolerance test (**B**) (*n* = 10) were measured in the 8th and 9th week during the experiment, respectively. (**C**) Curves of blood glucose levels and the calculated area under curve (AUC) (inner graph). Values are expressed as the mean ± SEM. (**D**) Serum concentrations of alanine aminotransferase (ALT), aspartate aminotransferase (AST), cholesterol (CHOL), high-density lipoprotein (HDL), low-density lipoprotein (LDL), and triglycerides (TGs) in mice (*n* ≥ 8). (**E**) Plasma concentrations of transforming growth factor alpha (TGF-α), interleukin-6 (IL-6) and lipopolysaccharide (LPS) in mice. Values are presented as the mean ± SEM.

We measured fasting serum glucose and insulin levels to determine the impact of CP on glucose homeostasis and insulin sensitivity. As shown in Figure 2A, after injection of glucose, mice administrated with HP supplement reversed their glucose level faster than mice who were untreated. This also occurred in male mice treated with LP. The corresponding area under curve (AUC) and HOMA-IR also demonstrated that HP administration improved glucose tolerance and insulin resistance in both female and male mice (Figure 2B,C).

We also examined the effect of CP and HFD on serum hyperlipidemia and found that it differed depending on the gender of mice. In male mice, HFD induced a significant increase in AST, ALT, CHOL, HDL and TG levels significantly (*p* < 0.05), and CP reduced AST, CHOL, HDL and TG level significantly (*p* < 0.05), but had little effect on ALT and LDL level (Figure 2D). In contrast, HFD only increased the ALT and HDL level in female mice, and CP administration did not change the concentrations of these markers.

Previous studies have demonstrated the interaction between inflammation and obesity via serum endotoxins and inflammatory cytokines [18,26,27]. Thus, we examined LPS, TNF-α and IL-6 concentrations in HFD-fed mice with or without CP. The mild systemic inflammation in HFD-fed mice was found effectively alleviated by CP (Figure 2E). A decreasing endotoxin (LPS) level was also found in CP-treated groups.

### 3.3. CP Prevented Liver Steatosis and Promoted Liver Lipid Metabolism in HFD-fed Mice

**Figure 3:**
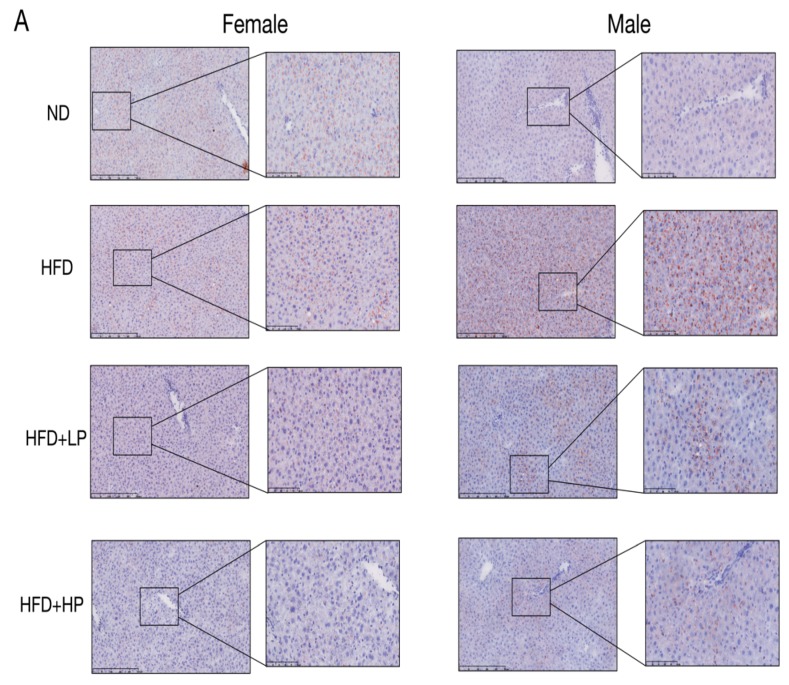

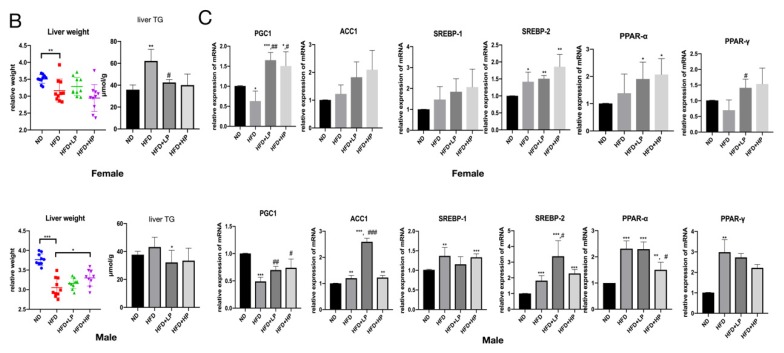
CP application prevented liver steatosis and promoted liver lipid metabolism in HFD-fed mice. (**A**) Liver Oil Red O staining of each group in both female and male mice. Each bar: 50 μm in original photo; 20 μm in enlarged photo. (**B**) Liver TG concentration and liver weight were changed according to the diet. (**C**) The relative expression of *PGC1*, *ACC1*, *SREBP1/2*, and *PPARɑ/γ* mRNA in liver, and were normalized with *GADPH* (*n* = 8). Values are presented as the mean ± SD.

Obesity is often accompanied with steatohepatitis [28]. CP treatment, even at a low concentration, effectively prevented lipid accumulation in liver tissue, demonstrable by Oil Red O staining in both female and male mice (Figure 3A). In addition, CP significantly (*p* < 0.005) reduced the liver weights of both female and male HFD-fed mice compared to controls. Further, HP reversed this in the male group (*p* < 0.05). The liver TG concentration also significantly declined, consistent with the beneficial effect of CP on liver steatosis (Figure 3B).

To examine the molecular pathways that regulate lipid metabolism, we measured mRNA expression of lipid metabolism-related genes in liver, including *PGC1*, *ACC1*, *PPARɑ/γ* and *SREBP1/2* (Figure 3C). HFD markedly enhanced the relative mRNA expression of *ACC1*, *PPARα/γ*, and *SREBP1/2*, and reduced the expression of PGC1 significantly in male mice. In female mice, only *PGC1* and *SREBP2* was regulated significantly by HFD. HP treatment restored *PGC1* expression in both female and male mice, and significantly decreased the PPAR-α level in male mice.

### 3.4. CP Promotes Browning of Adipose Tissue in HFD-fed Mice

**Figure 4:**
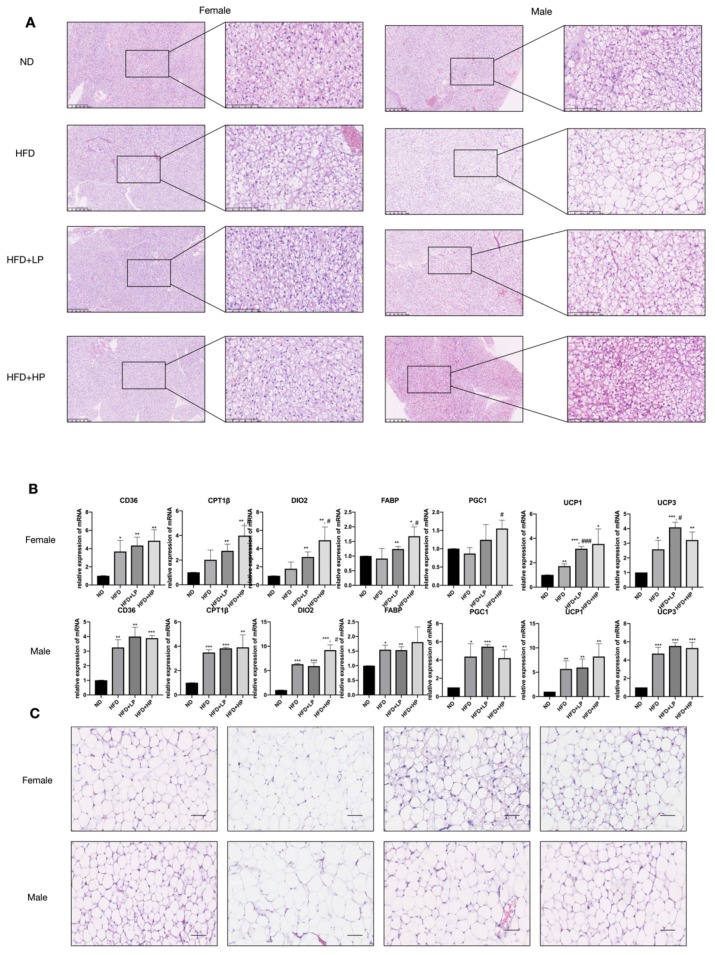
Effects of CP on adipose tissue (BAT & WAT) formation in HFD-fed mice. (**A**) Hematoxylin and eosin (H&E) staining of brown adipose tissue section. Each bar: 50 μm in original photo; 20 μm in enlarged photo. (**B**) The relative mRNA expression of adipose browning-related genes (*CD36, CPT1β, DIO2, FABP, PGC1, UCP1,* and *UCP3*) in BAT. (**C**) H&E staining of epididymal and parametrial white adipose tissue sections. Scale: 100 μm.

HFD enhanced the lipid accumulation in mice, which is considered as “whitening” of the BAT, especially in male mice (Figure 4A), while CP treatment (LP and HP) successfully reversed the lipid accumulation and stimulated the growth of BAT (“browning”). HFD increased the size of adipocyte cells collected from Epi-WAT and Par-WAT section (Figure 4C), while the size remained similar to the ND group and propolis-fed groups. Hence, we examined the mRNA expression levels of mitochondrial biogenesis (*PGC1α*), energy expenditure (*DIO2*), fatty acid catabolism (*CPT1β*), lipid transport (*CD36* and *FABP*) and thermogenic genes (*UCP1* and *UCP3*) in BAT. As shown in Figure 4B, all genes were elevated significantly in HFD-fed male mice, while only *CD36*, *UCP1* and *UCP3* increased in female mice. Oral administration with CP increased all the markers expression levels compared to the ND group, regardless of gender, and the CP supplementation group had higher levels of *CPT1β*, *DIO2*, *FABP*, *PGC1* and *UCP1/3* in comparison to the HFD group in female mice (*p* < 0.05). In male mice, only *DIO2* expression level was significantly elevated in CP administration group compared to the HFD-fed group. We observed no differences in *CD36*, *FABP*, *PGC1* and *UCP1/3* expression levels. Collectively, these results demonstrated that CP stimulates BAT browning and thermogenesis in HFD-induced obese mice.

### 3.5. The Effects of CP on Gut Microbiota Alteration

**Figure 5:**
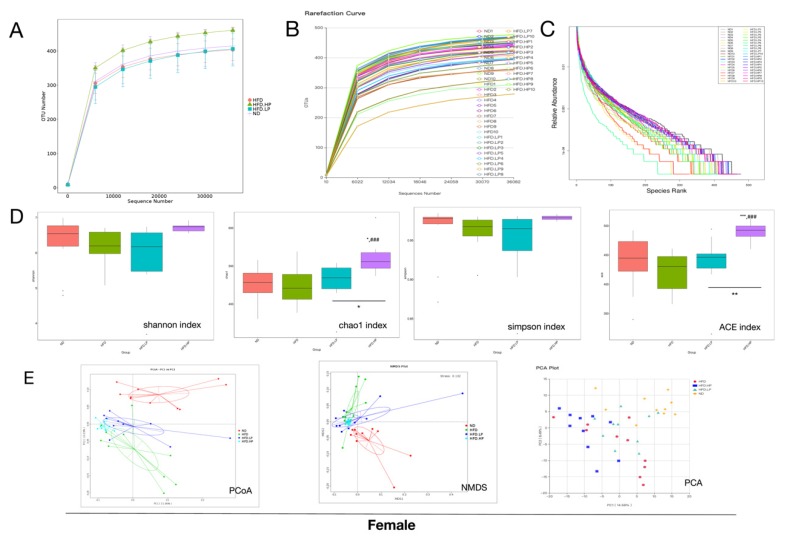

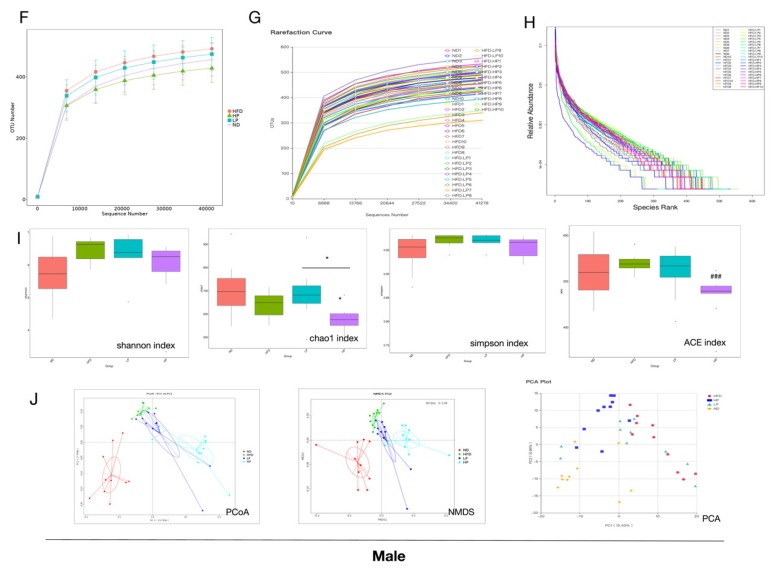
CP application altered microbial diversity and structure in HFD-fed mice. The operational taxonomic unit (OTU) rarefaction curve and the rank curve of microbial diversity responded to dietary change and CP treatment in female mice (**A**–**C**) and male mice (**F**–**H**). (**D**,**I**) show the Shannon, Chao1, Simpson and ACE indexes in both genders. (**E**,**J**) Principal coordinate analysis PCoA score plot based on a binary Jaccard, non-metric multidimensional scaling (NMDS) score plot based on an unweighted, and principal component analysis (PCA) score plot based on weights.

Recent research has revealed the interaction between gut microbiota and obesity [21]. Thus, we determined whether the anti-obesity effect of CP modulates gut microbiota by sequencing the bacterial 16S rRNA V3 + V4 region. In female mice, a total of 3,076,876 raw reads were collected from 40 stool samples, and 2,895,343 clean reads were assessed after quality filtering, with an average of 72,383 per mouse. In male mice, a total of 3,334,760 raw reads were collected from 40 stool samples, and 3,144,620 clean reads were assessed after quality filtering, with an average of 78,616 per mouse. Microbial diversity differed among the ND, HFD, HFD+LP and HFD+HP groups (Figure 5). Interestingly, changes in the alpha diversities of gut microbiota differed by gender according to the rarefaction and rank curves (Figure 5A). In female mice, HFD reduced the alpha diversity of intestinal microbiota. In contrast, in male mice, HFD increased the alpha diversity of intestinal microbiota. In both genders, a low concentration of CP administration failed to influence the microbiota diversity, while a high concentration of CP reversed the change made by HFD in both female and male mice. We also calculated the Shannon, Chao 1, Simpson, and ACE indexes. Consistent with the number of operational taxonomic units (OTUs), a high concentration of CP significantly increased the richness and diversity of gut microbiota in female HFD-fed mice, but had the opposite effect in male mice. In order to investigate the structural changes in intestinal microbiota, we used non-metric multidimensional scaling (NMDS), principal coordinate analysis (PCoA), and principal component analysis (PCA). Based on PCoA and NMDS results, there was a distinct clustering of gut microbiota within different diet groups in both female and male mice.

### 3.6. Relative Abundance of Gut Microbiota Changes in Response to HFD and CP Treatment

**Figure 6:**
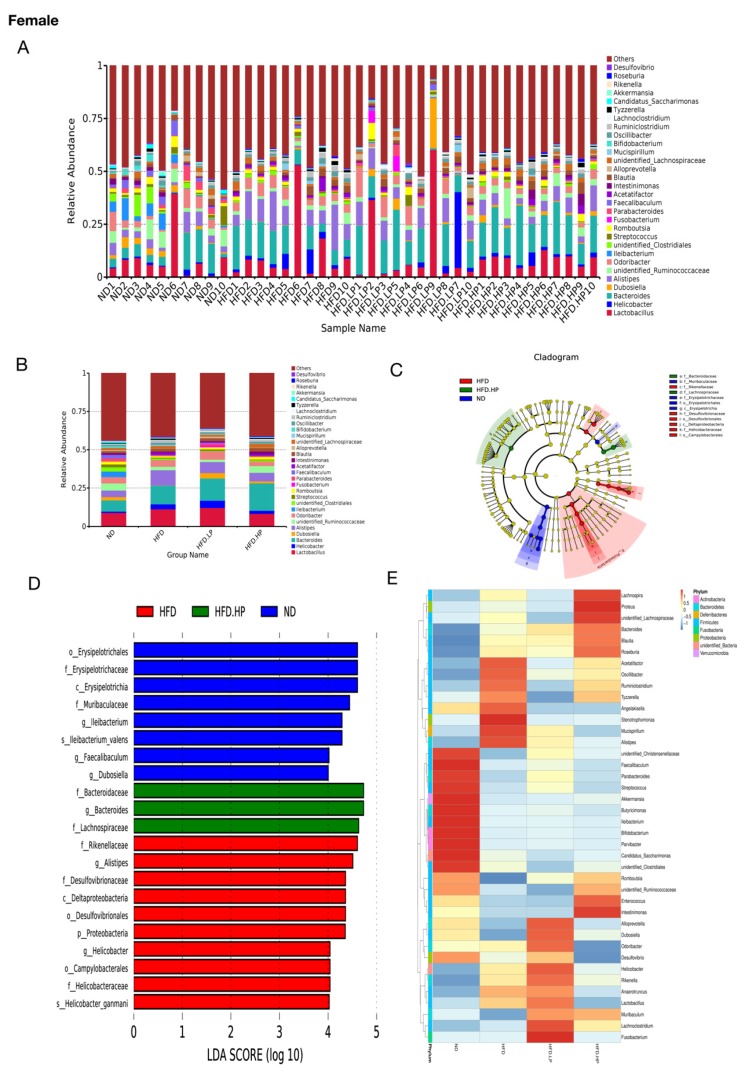

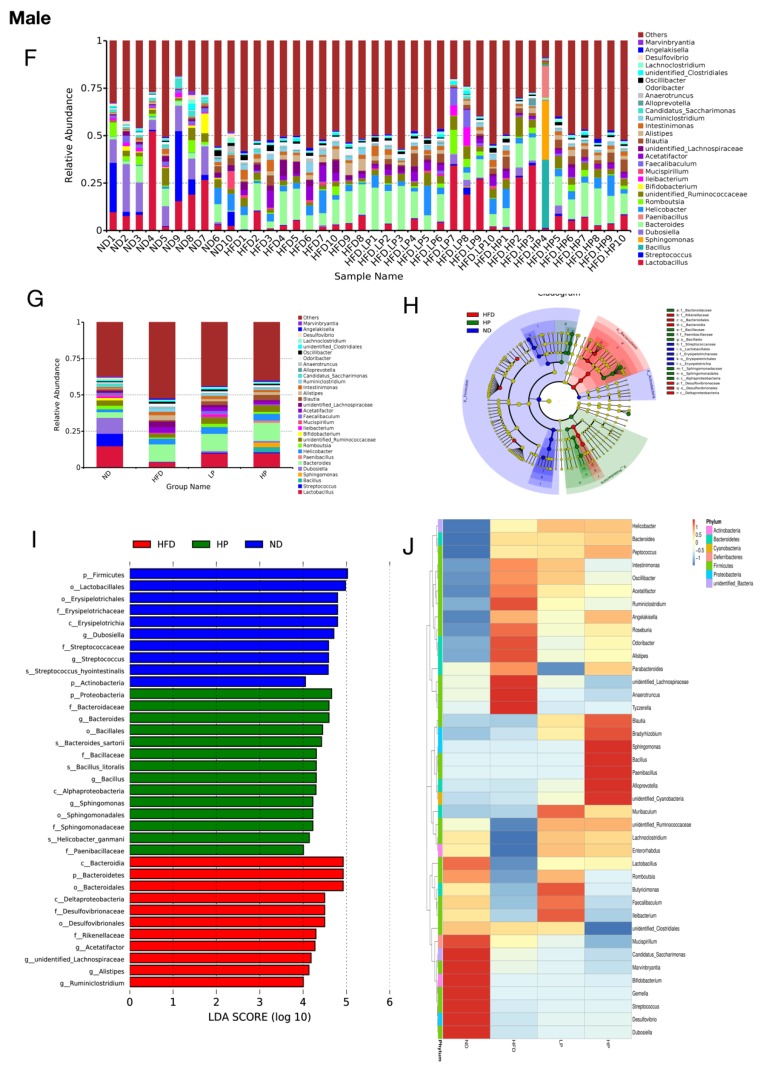
Differences in the bacterial community induced by diet and treatment according to the relative abundances of gut microbiota at the genus level and LEfSe analysis. (**A**,**B**,**F**,**G**) The relative abundance of intestinal microbiota at the genus level in the four groups of both genders. (**C**,**D**,**H**,**I**) LEfSe results showed the change in microbiota under HFD and HP administration. The LDA significant threshold was 4.0. Red, green, and blue represented HFD, HP, ND, respectively. (**E**,**J**) Heat maps of taxons that were most significantly different in abundance between the four groups at the genus level generated by LEfSe analysis.

Previous research has demonstrated that obesity is accompanied by changes in gut microbiota structure [17]. At the phylum level, *Bacteroidetes* was increased, while *Firmicutes* was reduced in the HFD group of either gender. However, there was a difference between female and male mice at the genus level. In female mice, the HFD group had a higher level of *Lactobacillus* (Figure 6A,B). In contrast, *Lactobacillus* was down-regulated significantly in male HFD-fed mice (Figure 6F,G), and ingestion of CP restored the level of *Lactobacillus* in male mice even in the LP group. In contrast, the changes in *Lactobacillus* in female mice induced by HFD were only reversed in the HP group. A similar trend was observed in *Alistipes* in both female and male mice. HFD increased *Alistipes*, and CP reversed the trend in a dose-dependent manner. Except for the bacteria mentioned above, the levels of *Bacteroides* were also abundant in the HFD groups of both genders.

We also used Linear discriminant analysis effect size (LEfSe) to identify the taxa associated with obesity and CP treatment. A total of 12 taxa in female and 18 taxa in male showed significant differences in the relative abundance among the ND-, HFD- and HP-treated groups (Figure 6C–E,H–J). In both genders, the ND group had more bacterial class *Erysipelotrichia*, whereas the HFD group had more *Deltaproteobacteria*. In female mice, the families *Rikeneliaceae*, *Desulfovibrionaceae* and *Helicobacteraceae* were also more abundant in the HFD group than the ND group. In the HP group, the families *Bacteroidaceae* and *Lachnospiraceae* were increased compared to the HFD group. Interestingly, male mice fed HP also had increased *Bacteroidaceae*.

### 3.7. Effects of CP on SCFA Production

**Figure 7:**
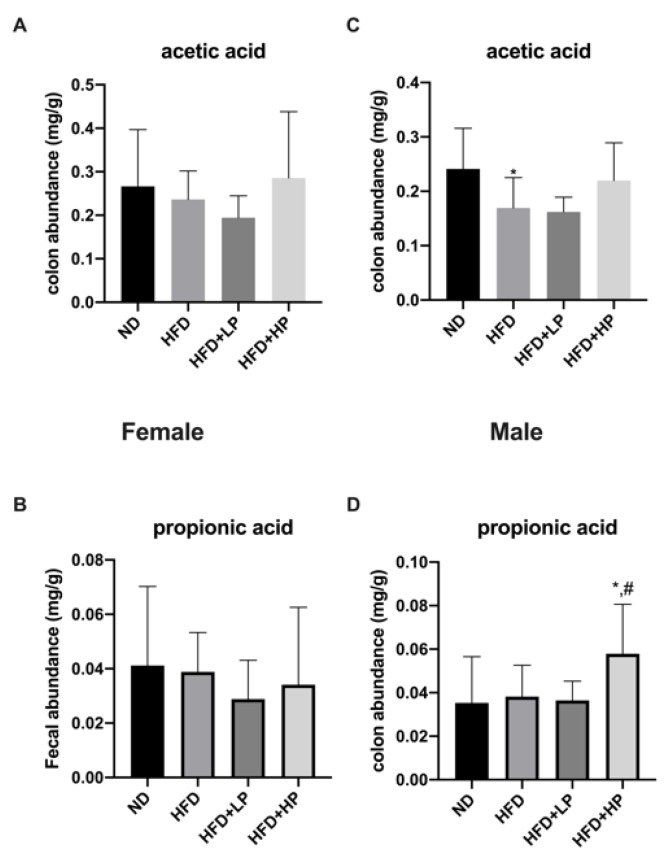
The modulation of main colon short-chain fatty acid (SCFA) concentrations responded to HFD and CP administration in female mice (**A**,**B**) and male mice (**C**,**D**). Data are presented as the mean ± SD.

To further examine the effect of CP on gut health, we analyzed short-chain fatty acid (SCFA) concentration in the colon, including acetic acid, propionic acid, isobutyric acid, butanoic acid and isovaleric acid. Acetic acid and propionic acid were the two most abundant SCFAs in both genders, while other acids were present at less than 0.01 mg/g (data not shown). There was no significant change in acetic acid and propionic acid among groups in female mice, while acetic acid was reduced significantly in the HFD group in male mice. HP treatment showed a trend towards an increase in the concentration of acetic acid in male mice. Concentrations of propionic acid were also unaffected by HFD in male mice. Interestingly, the HP group showed the highest level of propionic acid among all the groups (Figure 7).

## 4. Discussion

The increasing incidence of obesity poses a great threat to public health worldwide. The close association between obesity and multiple diseases contributes to increased morbidity and mortality [7,29]. Given that the consumption of plant-derived foods rich in polyphenols has positive effects on protecting against metabolic syndromes, these foods have been tested on obesity models [9,10]. Here, we studied the impact of CP on obesity development including glucose tolerance, lipid accumulation and gut microbiota alteration in both female and male mice models.

We found that CP administration prevented HFD-induced weight gain and fat mass accumulation in both female and male mice. Brown adipose tissue is characterized as a site for adaptive thermogenesis activated by the sympathetic nervous system, which is believed to be artificially induced [30]. In our study, CP administration reversed the “whitening” process induced by HFD in BAT and increased the expression of *UCP1* and *UCP3* in both female and male mice. These results suggest that CP can boost thermogenesis by promoting lipid metabolism and BAT growth in HFD-induced obese mice.

Furthermore, propolis prevented lipid accumulation and serum TG metabolism in male mice, although no significant difference was found in female mice. CP also improved glucose tolerance and insulin resistance in HFD-fed mice of both genders. The is consistent with previous studies reporting that dietary propolis can improve insulin sensitivity in the early stage of insulin resistance developed in different mice models [31,32,33], suggesting that the effect of propolis on glucose control is universal.

Accumulating evidence has suggested that gut microbiota play a significant role in disease development, including obesity, type 2 diabetes, and cancers [17,20,34]. Given that much still remains unknown about the process of phenolic phytochemical absorption after ingestion, it has been proposed that it may act primarily via the intestine [35,36]. Our results showed that HFD induced a shift in gut microbiota of mice. Interestingly, HFD was reported to increase the ratio of *Firmicutes* and *Bacteriodetes* in other studies [37,38], while lower *Firmicutes* and *Bacteriodetes* ratios were found in the HFD groups in mice of both genders in the present study. It should be noted that Schwiertz et al. also reported lower ratios of *Firmicutes* and *Bacteriodetes* in obese people [39]. Since the development of obesity and metabolic disorders is a complex process involving genetic and environmental factors, the genetic background of animal models and formations of forage potentially influence components of the gut microbiome. Furthermore, Arumugam et al. demonstrated that enterotypes in the human gut microbiome are more accurate, indicating that studies should not only focus at the phylum level [40]. Therefore, we also examined microbiota modulation at the genus level. *Alistipes*, a genus in the phylum *Bacteroidetes*, has been found to be abundant in humans with type 2 diabetes and obesity [22,41]. In our study, *Alistipes* increased significantly in both female and male HFD-fed mice. This was consistent with the uptrend of *Bacteroidetes* at the phylum level. Another genus that we addressed was the beneficial bacteria *Ruminococcaceae,* which decreased significantly in HFD-fed mice. *Ruminococcaceae* is one of the most abundant *Firmicute* families in the gut environment and is primarily responsible for digesting polysaccharides and fibers [42,43]. Its low concentration is positively related to NAFLD and increases intestinal permeability [44,45]. CP application was found to stimulate *Ruminococcaceae* growth, evidenced by an increasing abundance and thicker intestinal mucosa (Figure S1). This result suggests that CP ingestion was able to reverse the trend induced by HFD and improve gut microbiome structure in a dose-dependent manner, helping to improve intestinal health.

Chronic low-grade systemic inflammation is increasingly being recognized for playing a role in glucose, lipid metabolism in the early stage of type 2 diabetes and obesity pathogenesis [27,46]. It was also reported that inflammation contributes to NAFLD progression via elevated endotoxin levels [47]. Studies have revealed that infusion with LPS, known as endotoxins, elicited a similar inflammatory response as HFD in animals [48]. In the present study, CP gavage showed great ability to prevent NAFLD by reducing liver lipid accumulation and alleviating hepatic inflammation, which we believe is associated with the great capacity of CP to scavenge free radicals. Additionally, CP gavage reduced the LPS concentration significantly, as well as the circulating IL-6, IL-10 and TGFα concentration, demonstrating its anti-inflammatory effect in HFD. LPS and associated inflammatory molecules will spread into the bloodstream when gut permeability is modulated [48,49]. Further, the circulating LPS is mainly derived from Gram-negative bacteria in the intestinal ecosystem. In our study, *Alistipes*, the main producer of LPS, were found to be increased in HFD mice. However, CP ingestion reversed this increase. Furthermore, given that probiotic *Lactobacillus* alleviates LPS-induced inflammation and decreases TGFα level in vivo and in vitro [50,51,52,53], it is suggested that the increasing *Lactobacillus* may play a role in CP suppressing LPS-induced inflammation. Another researcher has pointed out that patients under an inflammatory environment usually had an increased TG level [54], which was in agreement with our result. The CP gavage effectively promoted TG metabolism in male mice (*p* < 0.05). Collectively, these results indicated that CP exerted a protective effect on the intestine by reducing *Alistipes* abundance, leading to lower inflammation and endotoxin levels in vivo.

In addition to establishing the intestinal environment, gut microbiota also stimulates epithelial regeneration by producing short-chain fatty acids (SCFAs) [55]. Interestingly, *Lactobacillus*, *Bacteroides* and *Alistipes*—all of which changed in this study—belong to acetic acid-producing genera. Yin et al. reported that there is a marked correlation between acetic acid production and *Bacteroides,* as well as *Alistipes* relative abundance [56]. In this study, HFD reduced acetic acid levels in colons, especially in male mice (*p* < 0.05). Further, HP application seemed to upregulate acetic acid levels, although there is no statistical significance between the two. We assumed that the modulation of intestinal microbiota induced by CP may also have an impact on SCFAs. Further experiments could be carried out to determine the interplay between SCFAs and CP administration.

Previous studies have proposed that sex could influence the efficacy of drugs [37,57], and experiments limited in male mice may not be able to provide thorough information about the function of drugs. In our study, we noticed that the composition of gut microbiota completely changed in HFD diet-fed male mice while the main microbiota remained similar in female mice. We also found that the anti-obesity effect of CP was gender related. Male mice were determined to be more sensitive to the CP treatment, as revealed by a lower working concentration of CP. As for the fat development induced by HFD, CP shows better performance in male mice than female mice according to the metabolic parameters and fat mass data (Figure 1 and Figure 2B). Gelineau et al. has put forward that female mice were more resistant to HFD-induced physiological changes, showing lower levels of insulin secretion and better glucose tolerance in comparison to male mice, which may provide an explanation for our result [58]. Adipose tissue is also known to express and secrete a variety of metabolites, hormones, and cytokines that have been implicated in the development of insulin resistance and glucose tolerance [29,59]. As expected, CP improved post glucose hyperinsulinemia in both female and male HFD-fed mice, which is supported by a lower fasting glucose level and thus HOMA-IR index. However, CP failed to improve insulin resistance in female mice. We also found that *Lactobacillus* showed the opposite trend in female and male HFD-fed mice. Although diet is believed to be the primary factor driving gut microbial changes, there is still evidence that these changes are sex dependent [60,61]. In a previous study, the probiotic strain *Lactobacillus* was proven to benefit intestinal immunity [62]. Its strains, *Lactobacillus johnsonii* La1 and *Lactobacillus gasseri* BNR17, can prevent weight gain under HFD and improve insulin sensitivity [51,63]. These findings were perfectly consistent with the changes in male mice. Further, we found that CP obviously restored the *Lactobacillus* level in HFD-fed male mice at both low and high concentrations. In contrast to male mice, *Lactobacillus* slightly increased in female HFD-fed mice, differing from previous studies. However, it should be noted that all previous studies about *Lactobacillus* only analyzed male mice. Additionally, Lee et al. discovered that the composition of vaginal microbiota is altered significantly by menopausal status, suggesting the composition of the human vaginal microbiota is highly associated with hormone levels [64]. Interestingly, recent studies have uncovered an interaction between estrogens and the microbiome that may affect metabolic rate, body weight, and adiposity [65]. E2 treatment could regulate the inflammation induced by HFD and increase the thickness and keratinization of epithelia [66]. Polyphenols were reported as ligands of estrogen receptors (ERs) due to their ER binding affinity, and are able to target hormone-related cancers [67]. Furthermore, emerging evidence shows that propolis has an estrogenic effect through activation of ERs [68,69]. Therefore, our results for female mice may support the idea that the microbiota is affected by the hormone status of the mice, especially by estrogen. Further experiments could be conducted to investigate the relation between estrogen level and CP treatment in HFD-fed mice. Collectively, our study indicated that the microbiota changes in HFD were sex dependent, and the mechanism underlying the anti-obesity effect of CP may be related to different hormone levels in female and male mice.

## 5. Conclusions

In summary, we reported for the first time that CP treatment could alleviate HFD-induced obesity. This effect was proven by less weight gain, better insulin sensitivity, and lower levels of metabolic endotoxemia, liver steatosis, and systemic inflammation. Furthermore, CP administration modulated gut microbiota by decreasing *Alistipes* abundance, regardless of gender, and increasing *Lactobacillus* in male mice. We believe that these two bacteria are playing an important role in the preventive effect of CP on obesity and type 2 diabetes. In addition, our results also support the theory that the composition of gut microbiota and responses to drugs differ by sex [37]. The effect of CP is more obvious in male mice, as compared to female mice. Collectively, Chinese propolis, which is rich in polyphenols, may prevent obesity and metabolic syndromes induced by diet, indicating the possibility of using CP as an alternative method for weight control.

## Supplementary Materials

The following are available online at https://www.mdpi.com/2072-6643/12/4/959/s1, Table S1: The components identified in CP, Table S2: The primers we use in this study, Figure S1: H & E-stained colon sections.
