# Supplementary material for: Chinese Propolis Prevents Obesity and Metabolism Syndromes Induced by a High Fat Diet and Accompanied by an Altered Gut Microbiota Structure in Mice
Source: Nutrients. 2020 Mar 30;12(4):959. doi: 10.3390/nu12040959 (PMC7230861; doi:10.3390/nu12040959)
Supplement: Supplementary file 1 [file nutrients-12-00959-s001.pdf]

nutrients

MDPI

Supplementary Materials:

Table S1. The components identified in CP.

Compounds	RT(min)	[M+1]$^+$	Content(mg/g)
Caffeic acid	8.26	181.04226	8.24
p-Coumaric acid	10.36	165.04734	4.97
Ferulic acid	11.35	195.05791	2.58
Isoferulic acid	11.87	195.05791	3.16
Cinnamic acid	12.21	149.05243	0.62
Vanillic acid	15.04	169.04226	\
Kaempferol	16.26	287.04774	1.72
Myricetin	16.38	319.03757	\
Quercetin	16.39	303.04265	1.75
3,4-dimethoxycinnamic acid	17.02	209.07356	9.26
Apigenin	18.41	271.05282	3.61
Pinobanksin	18.82	273.06847	35.99
Luteotin	18.90	287.04774	\
Caffeic acid benzyl ester	23.61	271.08921	\
Chrysin	23.66	255.05791	50.99
Pinocembrin	24.22	257.07356	43.93
Galangin	24.44	271.05282	18.24
CAPE	24.74	285.10486	15.79
3-O-Acetyl pinobanksin	24.91	315.07904	73.81
Rutin	32.10	611.15339	\

Table S2. The primers we use in this study.

Target gene	Sequence(5'-3')
	Real-time primers
ACC-1	F: GGACAGACTGATCGCAGAGA
	R: TGGAGAGCCCCACACACA
CD36	F: GGAACTGTGGGCTCATTGC
	R: CATGAGAATGCCTCCAAACAC
CPT1β	F: GGCACCTCTTCTGCCTTTAC
	R: TTTGGGTCAAACATGCAGAT
FABP	F: AGCATCATAACCCTAGATGGCG
	R: CATAACACATTCCACCACCAGC
FAS	F: CCCTTGATGAAGAGGGATCA
	R: ACTCCACAGGTGGGAACAAG
PGC1α	F: GGAGCCGTGACCACTGACA
	R: TGGTTTGCTGCATGGTTCTG
UCP1	F: TAAGCCGGCTGAGATCTTGT
	R: GGCCTCTACGACTCAGTCCA
UCP3	F: ATGAGTTTTGCCTCCATTCG
	R: GGCGTATCATGGCTTGAAAT
SREBP1	F: GAACGACATCGAAGACATGC
	R: GAGAAGCTCTCAGGAGAG
SREBP2	F: GTGCGCTCTCGTTTTACTGAAGT
	R:GTATAGAAGACGGCCTTCACCAA
PPARα	F：AGGCTGTAAGGGCTTCTTTCG
	R：GGCATTTGTTCCGGTTCTTC
PPARγ	F: CCATTCTGGCCCACCAAC
	R: AATGCGAGTGGTCTTCCATCA
DIO2	F: AATTATGCCTCGGAGAAGACCG
	R: GGCAGTTGCCTAGTGAAAGGT
GADPH	F: GTCGTGGATCTGACGTGCC
	R: TGCCTGCTTCACCACCTTCT

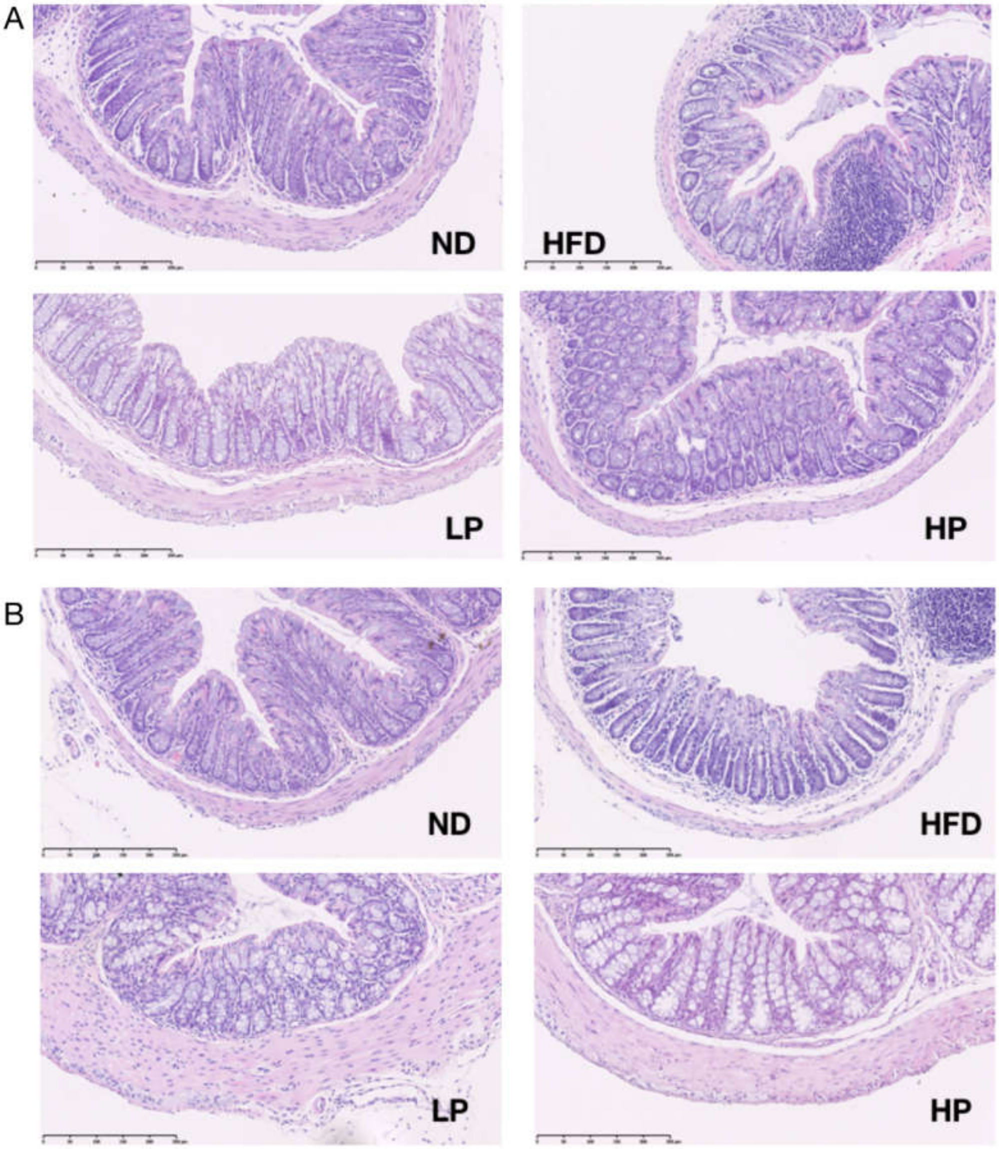

Figure 1. Hematoxylin and eosin (H & E)-stained colon sections.
